# A pilot randomized controlled trial of an integrative psychotherapy incorporating an attachment-based approach for adolescents with cannabis use disorder (TIMCA)

**DOI:** 10.1016/j.abrep.2026.100758

**Published:** 2026-10-10

**Authors:** Bou Nassif Yara, Richter Joachim, Kheirallah Lana, Farquhar Farrell, Gonnod Alix, Cros Carolina, Benyamina Amine, Varescon Isabelle, Rahioui Hassan

**Affiliations:** a Service des troubles du neuro-développement chez l'adulte, Groupe Hospitalier Universitaire Paris psychiatrie et neurosciences, Paris, France; b Laboratoire de psychopathologie et processus de santé, Université Paris Cité, Boulogne-Billancourt, France; c Service de psychiatrie et addictologie, Assistance Publique–Hôpitaux de Paris, Paris, France

**Keywords:** Adolescents, cannabis use disorder, Integrative psychotherapy, Integrated, attachment therapy, Motivational interviewing, Cognitive-behavioral therapy

## Abstract

**Background:**

Cannabis is the most used illicit substance among adolescents. Regular cannabis use is linked to cognitive and psychosocial difficulties. As no pharmacological treatment has proven effective, psychological interventions, including motivational interviewing (MI), cognitive-behavioral therapy (CBT), and family-based approaches, remain central. However, adolescent-specific evidence remains limited. TIMCA was developed as a ten-session integrative psychotherapy combining MI, CBT, and integrated attachment therapy. While family therapy may not be indicated for all adolescents, parental involvement remains important for the adolescent–parent relationship. TIMCA therefore combines individual and parent–adolescent sessions to address cannabis use alongside attachment-related and emotion-regulation processes.

**Objectives:**

This pilot randomized controlled trial evaluated the feasibility and acceptability of TIMCA and explored changes over time in cannabis use and relational, emotional, anxiety/depressive, and treatment-adherence outcomes compared with treatment as usual (TAU).

**Methods:**

Thirty-nine adolescents with cannabis use disorder were randomly assigned to TIMCA (*n* = 19) or TAU (*n* = 20) in two outpatient addiction services in the Paris metropolitan area. Cannabis use, attachment security, emotion regulation, anxiety, and depressive symptoms were assessed at baseline, mid-treatment, post-treatment, and 1-month follow-up using self-report measures and urine toxicology.

**Results:**

TIMCA was feasible and acceptable, with high treatment adherence. Exploratory analyses showed significant within-group changes, including reductions in cannabis use, emotion dysregulation, and depressive symptoms, along with improved attachment security. In TAU, within-group changes did not reach statistical significance, and no significant between-group differences were detected.

**Conclusion:**

TIMCA appears feasible and acceptable, supporting further evaluation in adequately powered trials with longer follow-up.

## Highlights

• TIMCA combines motivation, cognitive-behavioral, and attachment therapy.
• TIMCA is a 10-session therapy with individual and parent–adolescent sessions.
• TIMCA was feasible and acceptable for adolescents with cannabis use disorder.
• Within TIMCA, cannabis use and depressive symptoms decreased; attachment improved.
• Findings support further evaluation in a larger randomized trial.

## 1 Introduction

Cannabis is the most commonly used illicit psychoactive substance worldwide (United Nations Office on Drugs and Crime, 2023). In Europe, it is responsible for over two fifths of first-time admissions to drug treatment services (European Union Drugs Agency, 2026). France reports among the highest levels of cannabis use (CU) in Europe, particularly among adolescents and young adults, with 11% reporting use in the past month. In comparison, 9.6% of Europeans aged 15–24 years reported CU in the last month (European Union Drugs Agency, 2026). Use remains more frequent among males, although the gender gap is less pronounced in the youngest age groups (Spilka et al., 2024).

Cannabis use disorder (CUD), as defined in the DSM-5 (American Psychiatric Association, 2013), is a maladaptive pattern of CU leading to clinically significant impairment or distress, including loss of control, craving, social or occupational impairment, and continued use despite negative consequences. Adolescence is a period of profound neurobiological and psychosocial change, making adolescents particularly vulnerable to the effects of psychoactive substances such as cannabis that target the endocannabinoid system (Connor et al., 2021; Lichenstein et al., 2022). Early and regular CU has been associated with alterations in brain structure and function, cognitive difficulties such as impairments in attention and short-term memory, and poorer academic outcomes (Chan et al., 2024; Connor et al., 2021; Lichenstein et al., 2022). Longitudinal studies also link CU with increased risks of depression, anxiety, suicidal behavior, and psychotic disorders, particularly among those with preexisting vulnerabilities (Gobbi et al., 2019; Lichenstein et al., 2022; Penzel et al., 2021).

The development and maintenance of CUD reflect the interaction of biological, psychological, and social factors. Among these, attachment insecurity and emotion dysregulation have been identified as significant risk factors for CUD, particularly during adolescence (Fairbairn et al., 2018). According to attachment theory, attachment security and emotion regulation are intrinsically linked: when a stressor activates the attachment system, individuals seek proximity to an attachment figure (Bowlby, 1982). Sensitive and responsive parents can validate emotional experiences, provide comfort and help regulate distress, thereby fostering more adaptive emotion regulation (Mikulincer & Shaver, 2019). When these primary strategies are repeatedly unsuccessful, a sense of security may not fully develop, contributing to insecure attachment and negative internal representations of the self and others (e.g., feeling unworthy of love and/or perceiving others as untrustworthy) (Bowlby, 1982). Children may then adapt their attachment behaviors to the responses from the environment, leading to secondary strategies to manage attachment needs (Main, 1996). Hyperactivation involves amplifying distress and negative affect to maintain proximity to the attachment figure and obtain support, whereas deactivation involves minimizing and suppressing attachment needs, with greater emotional distance and avoidance of intimacy (Main, 1996; Mikulincer & Shaver, 2019).

During adolescence, increasing autonomy is accompanied by reduced time with parents and greater investment in peers and romantic relationships; however, parents remain primary attachment figures in times of distress, making relationship quality crucial (Allen et al., 2003; Moretti & Peled, 2004). Heightened emotional reactivity and the developmental need for independence may impede support-seeking, especially among adolescents with insecure attachment, who may also show interpersonal and metacognitive difficulties in emotionally charged situations (Allen, Grande, Tan & Loeb, 2018). Peer influences also contribute to CU trajectories (Barnett, DiGuiseppi, Tesdahl, & Meisel, 2021), but longitudinal studies suggest early CU and progression are primarily predicted by earlier family factors, including attachment security to parents and positive parenting practices (Leadbeater, Ames, Contreras, Thompson, & Goulet-Stock, 2022; Scholes-Balog, Hemphill, Heerde, Toumbourou, & Patton, 2020).

Psychosocial interventions remain the first-line treatment for CUD, as no pharmacological treatment has yet shown consistent efficacy (Nielsen, Gowing, Sabioni, & Le Foll, 2019). Approaches such as motivational interviewing (MI), cognitive-behavioral therapy (CBT), and family-based interventions are the most supported, but outcomes across trials are heterogeneous and often lack long-term durability.

Two studies provided the foundation for evaluating psychological interventions in adolescents with CUD. The Cannabis Youth Treatment (CYT) program in the United States compared five psychosocial interventions involving 600 adolescents. Treatment intensity ranged from brief MI/CBT to more intensive family- and community-based approaches. All approaches significantly reduced cannabis use, with no significant differences between treatments at one year (Dennis et al., 2004). In Europe, the INCANT study compared multidimensional family therapy (MDFT) with treatment as usual (TAU) in 450 adolescents across five countries, including France. Both therapies were delivered over six months (20–25 sessions for MDFT; 12–15 for TAU), with comparable effects on CU, internalizing symptoms, and family functioning, although MDFT showed greater benefits at 12-month follow-up among adolescents with more severe use or greater family conflict (Rigter et al., 2013; Schaub et al., 2014).

However, few studies have since specifically evaluated psychotherapy for adolescents with CUD, particularly in Europe. Most existing research focuses on broader substance use or brief preventive programs rather than clinical populations. Current recommendations emphasize the need for brief, integrative interventions tailored to adolescents, combining MI, CBT, and family components to address underlying mechanisms of CUD such as emotion regulation and interpersonal functioning (Adams et al., 2023; Bou Nassif, Rahioui, & Varescon, 2023; Connor, Manthey, Hall, & Stjepanović, 2025).

To address this gap, the Integrative Therapy for Adolescent Cannabis Misuse (TIMCA) was developed in a French university hospital setting as a ten-session individual psychotherapy that includes family sessions and integrates components of MI, CBT, and integrated attachment therapy (Bou Nassif, Hamonniere, Rahioui, & Varescon, 2024; Kheirallah, Belbeze, & Rahioui, 2025; Rahioui, 2016, Rahioui, 2026). This intervention aims to reduce CU, enhance adolescents' attachment security, and strengthen emotion regulation skills.

This pilot randomized trial evaluated the feasibility and acceptability of TIMCA and explored changes over time in cannabis use and relational, emotional, anxiety/depressive, and treatment-adherence outcomes among adolescents randomized to TIMCA or treatment as usual (TAU). Cannabis use was the main clinical outcome, while attachment security, emotion regulation, anxiety and depressive symptoms, and treatment adherence were examined as secondary exploratory outcomes to inform the design of a future fully powered trial.

## 2 Methods

### 2.1 Participants

Participants were recruited from two outpatient units specializing in adolescent addiction care within public hospitals in the Paris metropolitan area: the Adolescent Addiction Unit at Sainte-Anne Hospital, where recruitment took place from February 2023 to February 2025, with 30 participants recruited, and the Adolescent and Young Adult Unit at Paul-Brousse Hospital, where recruitment took place from February 2024 to February 2025, with 9 participants recruited. Participants were recruited during routine consultations, with initial appointments initiated by the adolescent, a parent/guardian, or a referring healthcare professional. A flyer summarizing the study objectives was also provided.

Eligible participants were adolescents aged 14 to 19 years and 11 months who met DSM-5 criteria for CUD (mild, moderate, or severe) and had at least one parent willing to participate actively in therapy sessions. Written informed consent was obtained from each participant and, when applicable, from their legal guardians.

Exclusion criteria included untreated current mood disorder, substance use disorder other than cannabis or tobacco, psychotic disorder, or current engagement in another psychotherapy. Participants were withdrawn if they were unable to continue outpatient therapy during the study period (e.g., hospitalization).

### 2.2 Procedure

This prospective multicenter randomized pilot trial employed a parallel two-arm design (1:1 allocation) comparing the effects of the TIMCA intervention with TAU. After verification of eligibility criteria and written informed consent, participants were randomly assigned to one of the two study arms through the REDCap platform. The randomization sequence was computer-generated from a pre-established list, stratified by site and sex, and balanced using variable block sizes. Ethical approval was granted by the Comité de Protection des Personnes (authorization no. 2022-A02067–36) on November 23, 2022, and October 5, 2023. The trial was preregistered on ClinicalTrials.gov (identifier NCT05765409) in accordance with international standards for transparency and trial reporting. Two interim analyses were prespecified after 30 and 60 participants completed follow-up, before completion of the planned sample. The present study reports the first interim analysis, after which the trial was paused for independent review to determine whether continuation was warranted.

### 2.3 Treatment conditions

All therapies were delivered by licensed clinicians with at least three years of experience in addiction care and trained in evidence-based interventions for substance use disorders. TIMCA was delivered by two therapists (a psychologist and a psychiatrist), both of whom were involved in the development of the intervention, whereas TAU was delivered by three clinical psychologists in the participating centers. Both conditions comprised ten weekly sessions.

#### 2.3.1 Intervention group (TIMCA)

TIMCA was a manualized intervention combining individual and family sessions that integrated elements of MI, CBT, and integrated attachment therapy (Bou Nassif et al., 2024; Rahioui, 2016, Rahioui, 2026). Intervention fidelity was monitored after each session using a 5-point Likert scale completed by the therapist who delivered the session, based on predefined session objectives and therapeutic targets.

Sessions grounded in MI and CBT, inspired by the CYT program (Dennis et al., 2004), focused on exploring ambivalence, strengthening motivation for change, conducting functional analyses of CU, and developing relapse-prevention and emotion regulation strategies.

Integrated attachment therapy sessions framed CU as an expression of emotional distress associated with adolescent developmental challenges and unmet attachment needs, emerging during the attachment transition from reliance on primary caregivers toward greater autonomy and increased investment in peer and romantic relationships (Rahioui, 2026). Family sessions involved both parents from the outset, emphasizing their role as primary attachment figures and their capacity to support recovery by responding to the adolescent's attachment needs and providing a secure base. Given the brief format, the intervention prioritized building a shared, coordinated caregiving system around the adolescent rather than addressing each parent's caregiving patterns separately, which would require more time.

Individual sessions aimed to clarify the adolescent's emotional reactivity in relation to CU. This involved identifying situations involving an attachment figure that activated the attachment system and the secondary attachment strategies mobilized (hyperactivation and/or deactivation), and examining how these patterns were associated with CU. The therapist then collaborated with the adolescent to develop and practice, through role-play, more adaptive strategies for regulating distress and expressing attachment needs in relationships with parents and peers (see Table 1).

**Table 1:** Overview of the TIMCA therapeutic framework.

Session	Format	Therapeutic approach	Objectives
1	Family	Integrated attachment therapy	Establish the therapeutic relationship; explore family dynamics and the adolescent's difficulties
2	Individual	Motivational interviewing	Provide personalized feedback; explore ambivalence and set goals
3	Family	Integrated attachment therapy	Clarify the adolescent's attachment needs; strengthen a shared, coordinated caregiving system around the adolescent
4	Individual	Cognitive-behavioral therapy	Conduct a functional analysis of cannabis use
5	Individual	Integrated attachment therapy	Explore emotional reactivity in relation to a parent and its link with cannabis use
6	Individual	Integrated attachment therapy	Explore emotional reactivity in peer relationships and its link with cannabis use
7	Individual	Integrated attachment therapy	Develop relational skills with parents through role-play
8	Individual	Integrated attachment therapy	Develop relational skills with peers through role-play
9	Individual	Motivational interviewing / cognitive-behavioral therapy	Develop a relapse-prevention plan
10	Family	Integrated attachment therapy	Review gains and consolidate skills; promote a balance between parental support and autonomy support

#### 2.3.2 Control group (TAU)

Participants assigned to the TAU condition received standard outpatient psychotherapy provided in the clinical centers. Therapy followed an integrative approach combining elements of MI and CBT, reflecting usual clinical practice for adolescents with substance use disorders. Parental involvement was determined on a case-by-case basis according to the adolescent's clinical needs. Fidelity was also monitored after each session using the same 5-point Likert scale.

### 2.4 Assessments

*Diagnostic evaluation***.** Inclusion and exclusion criteria were assessed through a semi-structured diagnostic interview based on DSM-5 criteria using the French version of the Kiddie Schedule for Affective Disorders and Schizophrenia – Present and Lifetime (K-SADS-PL; Thümmler & Askenazy, 2018). The interview covered current and past episodes of mood, psychotic, attention-deficit/hyperactivity, and substance use disorders and included both an adolescent and a parent component.

*Cannabis use.* CU was assessed with the Timeline Follow-Back procedure (TLFB; Robinson, Sobell, Sobell, & Leo, 2014). This calendar method evaluates the frequency and quantity of cannabis consumed over the past 30 days. Participants retrospectively completed the calendar, recording CU for each day. Quantity was measured as the number of self-reported joints, with each joint corresponding to one unit of CU, including partial joints. All participants (100%) reported CU in the form of joints. The TLFB has demonstrated good psychometric properties in adolescent populations.

In addition, urinary THC was assessed using the NarcoCheck® Prédosage test (KAPPA CITY BIOTECH), which uses five urine strips detecting THC at thresholds of 18, 50, 150, 300, and 600 ng/mL. Urine THC screening was used to document recent cannabis exposure at study visits and to complement self-report, but it was not intended as a sensitive measure of change over the short follow-up period.

*Attachment to parents.* The Inventory of Parent and Peer Attachment was used to assess the quality of the parent–adolescent relationship (IPPA; Armsden & Greenberg, 1987; Vignoli & Mallet, 2004). The parent version includes 28 items rated on a 5-point Likert scale and yields three subscales (trust, communication, and alienation) and a global attachment security score. The French version has shown good internal consistency among adolescents aged 14–19 years (Cronbach's α = 0.85–0.89).

*Emotion regulation.* The Regulation of Emotions Questionnaire (REQ-2; Phillips & Power, 2007; Amaro, 2013) is a 21-item self-report measure evaluating functional and dysfunctional emotion regulation strategies across intra- and interpersonal domains on a 5-point Likert scale. Examples of internal-functional strategies include rethinking thoughts or goals and putting situations into perspective, whereas internal-dysfunctional strategies include dwelling on thoughts and feelings, suppressing feelings, and self-punishment. External-functional strategies include talking to someone about their feelings or asking for advice, whereas external-dysfunctional strategies include verbal or physical aggression or attempts to make others feel bad (Phillips & Power, 2007). The French version, validated among adolescents aged 12–20 years, demonstrated acceptable internal consistency (α = 0.66–0.76) and good convergent validity with the IPPA.

*Depressive symptoms.* The Beck Depression Inventory consists of 21 items assessing the intensity of depressive symptoms over the past two weeks on a 4-point Likert scale (BDI; Beck, Ward, Mendelson, Mock, & Erbaugh, 1961; Byrne, Baron, & Campbell, 1994). French validation studies in adolescents and young adults have reported high internal consistency (α = 0.80–0.92) and good test–retest reliability (*r* = 0.62–0.74).

*Anxiety symptoms.* The State–Trait Anxiety Inventory is a 40-item self-report questionnaire assessing both state and trait anxiety on a 4-point Likert scale (STAI-Y; Spielberger, Gorsuch, Lushene, Vagg, & Jacobs, 1983; Bruchon-Schweitzer & Paulhan, 1993). The French version has demonstrated good reliability in adolescent samples (α = 0.82 for trait anxiety, α = 0.77 for state anxiety; test–retest *r* = 0.71–0.85).

*Treatment adherence.* Adherence was measured by the mean number of sessions completed per participant out of the ten sessions scheduled in the treatment protocol.

Assessments were conducted at four time points: baseline (T0), mid-treatment (T1; fifth session), post-treatment (T2; tenth session), and four weeks after treatment completion (T3).

### 2.5 Statistical methods

Analyses were conducted using R (v. 4.3) with the *lme4*, *lmerTest*, *robustlmm*, *mice*, and *emmeans* packages. All analyses were conducted in a modified intention-to-treat sample, including randomized participants who provided at least one baseline clinical outcome assessment. Missing data (16.7%) were handled under a missing-at-random assumption; baseline CUD severity was negatively correlated with the number of sessions completed (*r* = −0.36, *p* = .030), suggesting that missingness may have been related to baseline clinical severity.

Multiple imputation by chained equations (MICE) was used to mitigate potential bias (Enders, Mistler, & Keller, 2016). Fifteen complete datasets were generated and pooled using Rubin's rules to account for imputation uncertainty (Rubin, 1987). Linear mixed-effects models for repeated measures examined group, time, and time × group effects while accounting for participant-level variability. When residuals were non-normal, a Yeo–Johnson transformation was applied (Yeo & Johnson, 2000); if assumptions remained unmet, robust mixed models were used to reduce the influence of outliers (Schielzeth et al., 2020). Estimated marginal means were used to derive exploratory pairwise contrasts for between-group differences and within-group changes over time, with Holm correction to control for multiple comparisons (Bono, Alarcón, & Blanca, 2021). All tests were two-tailed with α = 0.05.

## 3 Results

### 3.1 Sample characteristics

A total of 39 adolescents were randomized (24 males, 61.5%), with a mean age of 17.2 years (*SD* = 1.25). Participant flow through the study is presented in Fig. 1. Two participants withdrew immediately after randomization and provided only sociodemographic information, with no baseline clinical outcome data, yielding a modified intention-to-treat sample of 37 participants.

**Fig. 1:**
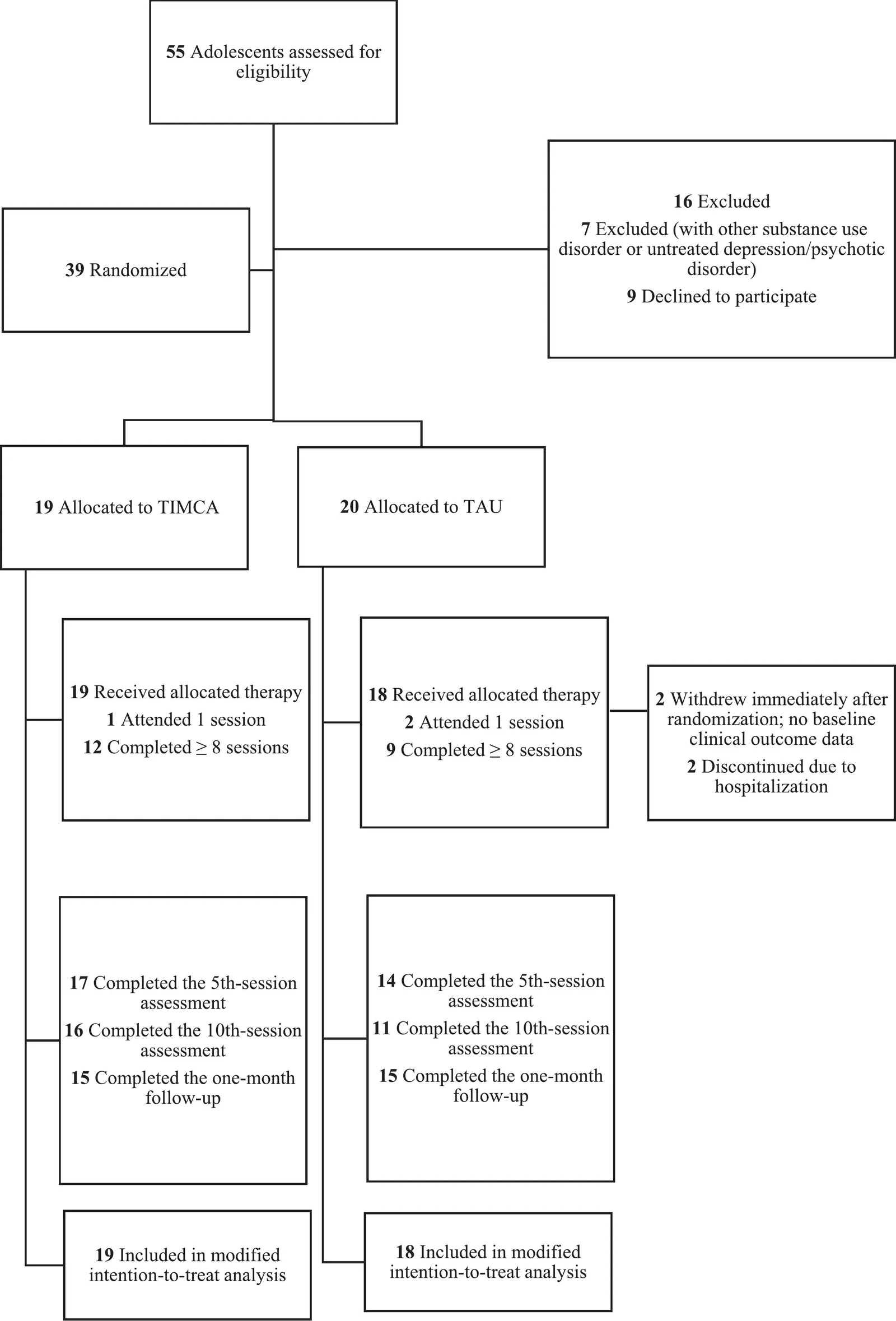
CONSORT flow diagram of participant flow through the study.

Regarding education or activity status, 66.7% of participants were currently enrolled in high-school or higher education. Among those not in school, 23.1% were involved in insertion or care programs. Concerning family structure, 30.8% lived with both parents, 43.6% in shared custody, and 23.1% with one parent.

At baseline, more than half of the sample (51.3%) met criteria for severe CUD. The mean age of first CU was 13.9 years (*SD* = 1.20), and the mean age for onset of regular use was 15.4 years (*SD* = 1.44). Most participants (59%) had already attempted to stop using cannabis, while 25.6% had previously received treatment for substance use.

Psychiatric comorbidity was common: 30.8% had been previously hospitalized, 53.8% reported a past major depressive episode, and 25.6% had a current stabilized psychiatric disorder. The most frequent current diagnoses were attention-deficit/hyperactivity disorder (25.6%) and mood disorders (7.7%), as assessed with the *K-SADS-PL* (DSM-5). At baseline, the TIMCA (*n* = 19) and TAU (*n* = 18) groups did not differ significantly on CU variables or secondary measures (attachment, emotion regulation, anxiety, and depressive symptoms), confirming the comparability of groups at inclusion. Participant sociodemographic and clinical characteristics are summarized in Table 2.

**Table 2:** Baseline sociodemographic and clinical characteristics of the randomized sample (*N* = 39).

Sociodemographic and clinical characteristics	Total sample (*N* = 39)
Male sex, %	61.5
Mean age (years) ± SD	17.2 ± 1.25
Education or activity status, %	
High-school education (general, technological, or vocational track)	51.3
Higher education (university, technical degree, or institute)	15.4
Insertion or care program (youth employment service, civil service, psychological follow-up)	23.1
No current activity (neither studying nor employed)	10.3
Family structure, %	
Living with both parents	30.8
Shared custody	43.6
Single parent	23.1
Cannabis use disorder severity, %	
Mild	23.1
Moderate	25.6
Severe	51.3
Mean age of first cannabis use (years) ± SD	13.9 ± 1.20
Mean age of regular cannabis use (years) ± SD	15.4 ± 1.44
Previous quit attempts, %	59.0
Prior substance-use treatment, %	25.6
Previous psychiatric hospitalization, %	30.8
Current psychotropic medication, %	33.3
Current psychiatric disorder, %	
Attention-deficit/hyperactivity disorder (ADHD)	25.6
Major depressive episode	10.3
Mood disorders (other than major depression)	7.7

Analyses of changes over time for the primary and secondary outcomes are reported below and summarized in Table 3. Several outcomes showed significant exploratory within-group improvements in the TIMCA group, while changes in TAU were generally non-significant. No significant between-group effects were detected in this pilot sample.

**Table 3:** Descriptive and inferential statistics for primary and secondary outcomes by group and time.

Variable (Scale range)	TIMCA T0 M (SD)	TIMCA T3 M (SD)	TAU T0 M (SD)	TAU T3 M (SD)	Within-group Z (*p*)
Days abstinent (last 30 days; 0–30)	4.1 (7.9)	10.3 (12.2)	4.9 (7.1)	10.8 (11.8)	−2.76 (0.006)** (T0–T2)
Total joints (last 30 days)	114 (61.0)	71.9 (73.2)	93.5 (71.9)	82.9 (103.0)	−2.48 (0.013)* (T0–T3)
Attachment security (IPPA; −20–92)	40.2 (23.0)	45.7 (19.5)	31.6 (22.2)	37.8 (19.6)	−2.02 (0.044)* (T0–T2)
Emotion regulation – internal dysfunctional (REQ-2; 5–25)	9.4 (5.0)	6.7 (3.7)	10.1 (3.1)	9.9 (3.1)	−2.48 (0.030)* (T0–T3)
Anxiety (STAI-Y; 20–80)	46.4 (10.2)	43.3 (13.3)	44.0 (11.3)	44.6 (8.2)	— (all *p ≥* .18)
Depressive symptoms (BDI; 0–63)	23.1 (14.0)	15.9 (12.5)	25.3 (12.0)	20.2 (11.8)	−2.48 (0.013)* (T0–T3)

**Note.** Values are means (SD). Analyses used mixed-effects models with multiple imputation in a modified intention-to-treat sample. No significant between-group effects were detected. Within-group statistics are presented for the TIMCA group. TIMCA = Integrative Therapy for Adolescent Cannabis Misuse; TAU = treatment as usual; IPPA = Inventory of Parent and Peer Attachment; REQ-2 = Regulation of Emotions Questionnaire; STAI-Y = State-Trait Anxiety Inventory; BDI = Beck Depression Inventory. * *p* < .05; ** *p* < .01.

### 3.2 Primary outcomes: Cannabis use

#### 3.2.1 Frequency of use

*Days abstinent (last 30 days).* Within groups, the TIMCA group increased abstinent days from T0 to T2 (*p* = .006) and from T1 to T2 (*p* = .001), with further improvement from T1 to T3 (*p* = .020) and a trend from T0 to T3 (*p* = .062). The TAU group showed no significant change (trend T0 to T3, *p* = .086) (see Fig. 2*).* Across assessment points, abstinent days increased more consistently in TIMCA, whereas the TAU pattern fluctuated.

**Fig. 2:**
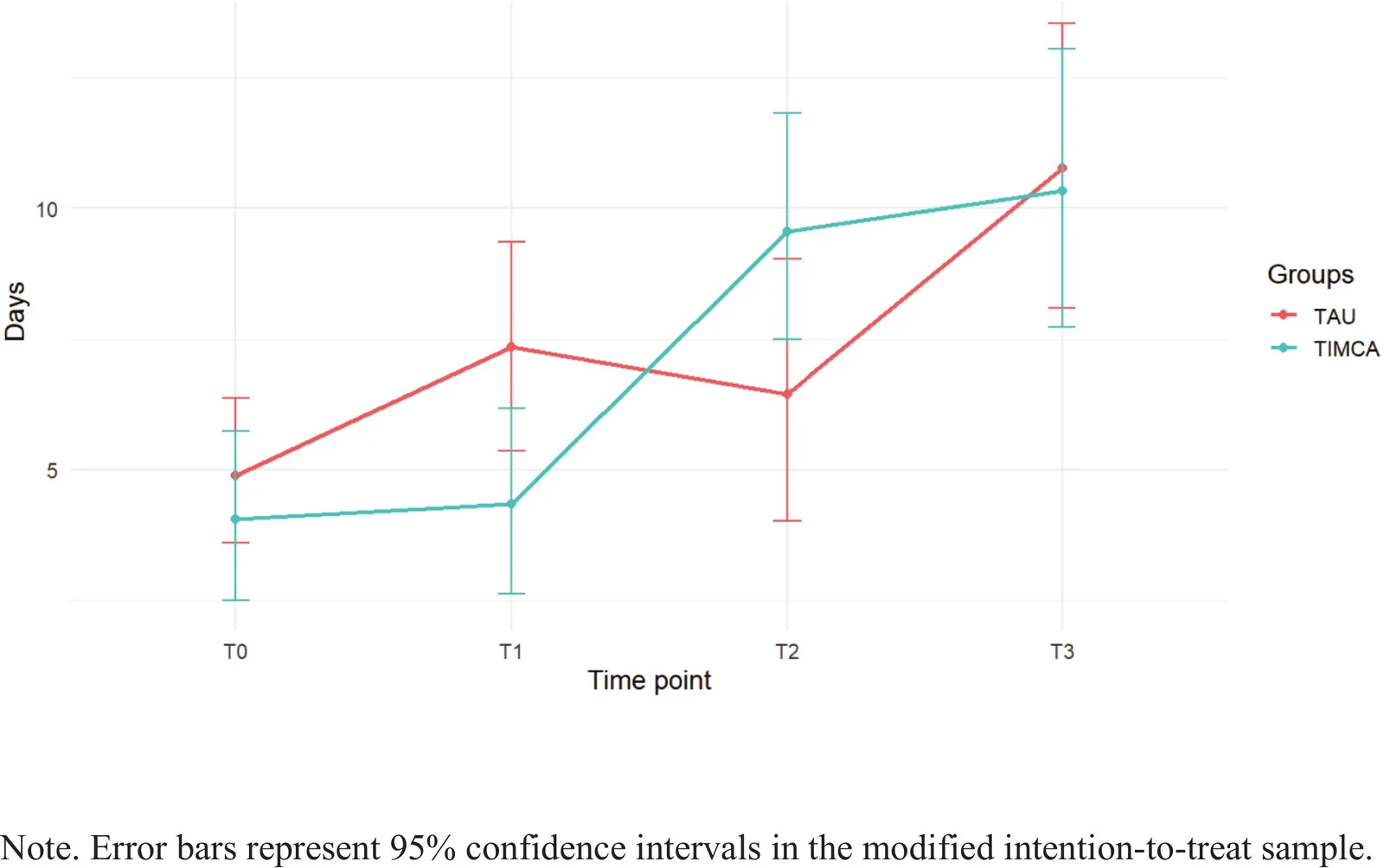
Changes in the total number of abstinent days (last 30 days) across assessment points (T0–T3) by group. Note. Error bars represent 95% confidence intervals in the modified intention-to-treat sample.

#### 3.2.2 Quantity of use

*Total cannabis joints (last 30 days).* Within groups, the TIMCA group reduced total joints from T0 to T2 (*p* = .037), from T0 to T3 (*p* = .013), and from T1 to T3 (*p* = .016), with a trend from T1 to T2 (*p* = .059). The TAU group showed no significant change (see Fig. 3). Across assessment points, the number of joints decreased more steadily in TIMCA, whereas the TAU pattern fluctuated.

**Fig. 3:**
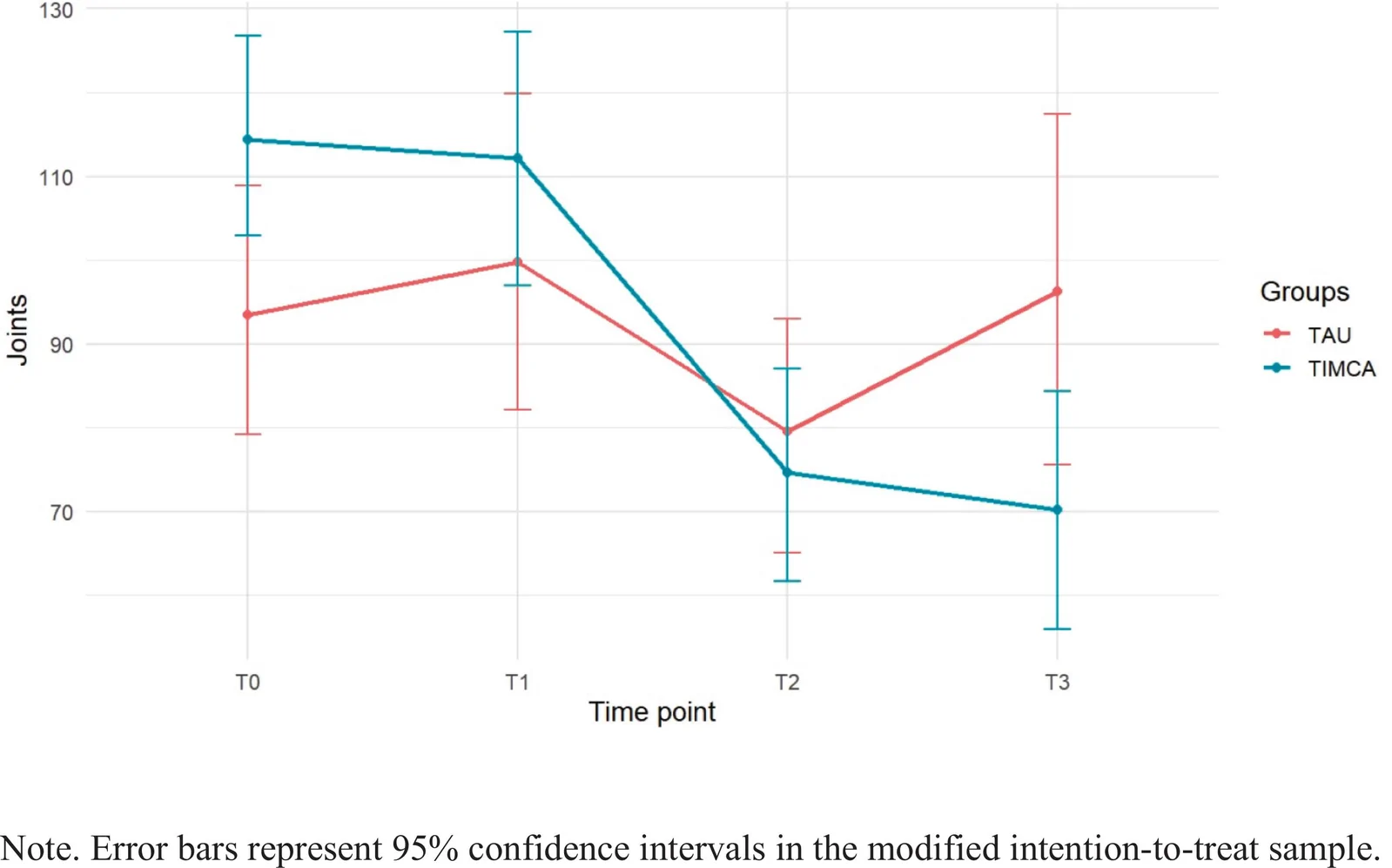
Changes in the total number of cannabis joints during the last 30 days across assessment points (T0–T3) by group. Note. Error bars represent 95% confidence intervals in the modified intention-to-treat sample.

*Urine toxicology.* Urine toxicology data were available for 82.1% of participants at baseline and 80.0% at the 1-month follow-up (T3). The objective urine screens did not show significant differences between or within groups at any time point.

### 3.3 Secondary outcomes: Attachment, emotion regulation, anxiety, and depressive symptoms

#### 3.3.1 Attachment security

A significant improvement was observed in the TIMCA group between T0 and T2 (*p* = .044) and a trend between T1 and T2 (*p* = .066). In the TAU group, no significant change was observed, although a nonsignificant trend emerged between T0 and T3 (*p* = .145).

#### 3.3.2 Emotion regulation

For external strategies, no significant changes were observed in the TIMCA group (all *p* > .18 for dysfunctional; all *p* > .31 for functional) or in the TAU group (all *p* > .88 for dysfunctional; all *p* > .25 for functional).

For internal strategies, no significant changes were observed in functional strategies for either group (TIMCA: all *p* ≥ .26; TAU: all *p* > .61). In contrast, dysfunctional internal strategies decreased significantly in the TIMCA group from T0 to T2 (*p* = .041) and from T0 to T3 (*p* = .030), with a trend from T0 to T1 (*p* = .054). No significant change was observed in the TAU group (all *p* > .42).

#### 3.3.3 Anxiety symptoms

For state anxiety, no significant changes were observed in either the TIMCA group (all *p* ≥ .186) or the TAU group (all *p* ≥ .520).

For trait anxiety, no significant changes were observed in either the TIMCA group (all *p* ≥ .283) or the TAU group (all *p* ≥ .461).

#### 3.3.4 Depressive symptoms

In the TIMCA group, depressive symptoms decreased significantly between T0 and T2 (*p* = .013) and between T0 and T3 (*p* = .034). In the TAU group, no significant change was observed (all *p* ≥ .161).

#### 3.3.5 Treatment adherence

Treatment adherence was generally high in both groups. The mean number of sessions completed out of the ten planned was 7.67 (median = 8) in the TIMCA group and 6.61 (median = 7.5) in the TAU group. The correlation between group and number of sessions was not significant (*r* = 0.196, *p* = .252).

No therapist-related effects were observed on any of the study variables. For example, at T3, Spearman's correlations ranged from −0.26 to 0.14, all nonsignificant (*p* ≥ .174), suggesting that the results were not influenced by individual therapist characteristics.

## 4 Discussion

This pilot randomized controlled trial evaluated the feasibility and acceptability of TIMCA and explored changes over time in cannabis use and relational, emotional, anxiety/depressive, and treatment-adherence outcomes compared with TAU.

The present sample was characterized by a particularly severe clinical profile. More than half of participants met criteria for severe CUD, with a mean of 26 days of use per month, which is higher than levels reported in large international cohorts such as the INCANT trial (≈61 days per 90; Rigter et al., 2013) and the CYT trial (36–60 days per 90; Dennis et al., 2004). Adolescents also showed marked psychosocial vulnerability: only 67% were enrolled in school or vocational training, compared with 87% in CYT (Dennis et al., 2004) and 75% in INCANT (Phan et al., 2011), and nearly 70% experienced parental separation, compared with 56% in the overall INCANT sample. Psychiatric comorbidity was frequent, with over half reporting a past major depressive episode and one-third reporting a history of psychiatric hospitalization.

Despite the sample's clinical severity, adherence was high overall, with good engagement among participants receiving TIMCA, including attendance at family sessions. Follow-up completion was 76.9% (30 of 39) at the 1-month assessment, supporting the feasibility of participant retention through follow-up.

Exploratory analyses showed significant within-group reductions in CU, emotion dysregulation, and depressive symptoms, along with improved attachment security in the TIMCA group. In the TAU group, no within-group changes reached statistical significance.

Between-group differences in cannabis outcomes were not observed in this pilot sample. This pattern is consistent with major adolescent psychotherapy trials, in which several evidence-based interventions yield comparable reductions in CU, including CYT (Dennis et al., 2004) and INCANT (Schaub et al., 2014). However, longer follow-up may be particularly important in more severe profiles, as differential effects have emerged in favor of MDFT at later time points in INCANT (Rigter et al., 2013; Schaub et al., 2014).

Several aspects of TIMCA's design may have contributed to treatment engagement and the observed improvements. The attachment-based therapeutic relationship, with a differentiated focus on the adolescent and their parents, may have supported engagement by providing a safe haven and a secure base. This emphasis on parental involvement is also consistent with evidence highlighting its importance in psychosocial treatment for adolescents with CUD (Adams et al., 2023; Bou Nassif et al., 2023). Grounded in integrated attachment therapy, TIMCA targets secondary attachment strategies and attachment-related distress within interpersonal contexts that can maintain CU during adolescence (Rahioui, 2026). This focus is consistent with recent evidence showing that attachment insecurity strengthens the association between problematic CU and depressive symptoms (Gliksberg et al., 2026). Attachment security and emotion regulation, which are rarely assessed in cannabis treatment trials, may help elucidate therapeutic processes in future adequately powered studies, including whether changes in these domains are associated with changes in CU (Adams et al., 2023; Bou Nassif et al., 2023). Given their broader role in psychopathology and addictive behaviors, these processes may also represent therapeutic targets across other substance use and psychiatric disorders (Fairbairn et al., 2018; Kheirallah et al., 2025; Mikulincer & Shaver, 2019). The brief, structured, integrative format of TIMCA, combining individual and family sessions, is well suited to outpatient settings and may support implementation. TIMCA therefore aligns with current recommendations advocating psychotherapeutic interventions that address both substance use and psychosocial mechanisms involved in adolescent CUD, particularly attachment-related processes (Adams et al., 2023; Ariss & Fairbairn, 2020; Bou Nassif et al., 2023).

This study has some limitations inherent to its pilot nature. The small sample size (*N* = 39) limited statistical power and the precision of both within- and between-group estimates. The absence of significant within-group changes in TAU should therefore be interpreted cautiously. In addition, the active nature of TAU, which included MI and CBT also incorporated in TIMCA, may have reduced the contrast between treatment conditions. Intervention fidelity scores were not included in the statistical analyses because the small number of TIMCA therapists and the heterogeneous delivery of TAU across sites limited comparison of fidelity across therapists. TIMCA therapists were involved in the development of the intervention, raising the possibility of researcher allegiance (Dragioti, Dimoliatis, & Evangelou, 2015). The short follow-up period prevented assessment of long-term maintenance or relapse. Self-reported measures may be subject to recall and social desirability biases.

Future trials should therefore use larger samples, longer follow-up, independent therapists, and standardized fidelity monitoring. Assessing adolescents' and parents' experiences of TIMCA would provide further insight into parental involvement, particularly adolescents' comfort in discussing CU and related difficulties and parents' understanding of adolescents' CUD and responsiveness to their attachment needs. Despite these constraints, this study adds to the limited European evidence on psychotherapy for adolescents with severe CUD. The inclusion of adolescents with severe, comorbid psychiatric disorders and balanced gender representation further enhances the ecological validity of the study and its relevance to routine clinical populations.

## 5 Conclusion

TIMCA appears feasible and acceptable for adolescents with severe CUD and marked psychosocial vulnerability. To our knowledge, TIMCA is the first manualized individual psychotherapy for adolescents with CUD that explicitly targets attachment-related difficulties by addressing emotional and relational processes. Exploratory within-group changes in the TIMCA group included reductions in CU, emotion dysregulation, and depressive symptoms, with improvement in attachment security to parents. These preliminary findings warrant further investigation in larger, adequately powered trials with longer follow-up.

## CRediT authorship contribution statement

**Bou Nassif Yara:** Writing – review & editing, Writing – original draft, Methodology, Investigation. **Richter Joachim:** Validation, Formal analysis, Data curation. **Kheirallah Lana:** Writing – review & editing, Investigation. **Farquhar Farrell:** Writing – review & editing, Investigation. **Gonnod Alix:** Writing – review & editing, Investigation. **Cros Carolina:** Writing – review & editing, Investigation. **Benyamina Amine:** Writing – review & editing, Resources. **Varescon Isabelle:** Writing – review & editing, Supervision, Methodology. **Rahioui Hassan:** Writing – review & editing, Supervision, Resources, Project administration, Methodology, Investigation, Conceptualization.

## Ethics declaration

**Informed consent and patient details**: Written informed consent to take part in the study and to publish the article has been obtained from all participants or their legal representatives. The privacy rights of participants have been observed.

**Studies in Human**: This study was performed in compliance with relevant laws, regulatory frameworks and guidelines where the research took place. This study was approved by the Comité de Protection des Personnes. (Approval No. 2022-A02067–36)

**Clinical Trials**: The results of this clinical trial and any associated work have been posted in a registry. This clinical trial was registered with number NCT05765409 (ClinicalTrials.gov).

## Funding

The study received funding (€69,130) from an internal research grant of the GHU Paris on July 12, 2022.

## Declaration of competing interest

The authors declare that they have no known competing financial interests or personal relationships that could have appeared to influence the work reported in this paper.

## Data Availability

Data will be made available on request.
